# Maternal and Newborn Outcomes of SARS-CoV-2/COVID-19 and Pregnancy: Parallels and Contrasts with Human Immunodeficiency Virus/Acquired Immunodeficiency Syndrome

**DOI:** 10.18103/mra.v12i4.5205

**Published:** 2024-04-29

**Authors:** Dan Li, Jing Zhang, Xiaofen Zhang, Yifan Chang, Sten H. Vermund

**Affiliations:** 1. Yale School of Public Health, New Haven, CT 06511, USA; 2. Shunyi Maternal and Children’s Hospital of Beijing Children’s Hospital, Beijing 101300, China

**Keywords:** COVID-19, SARS-CoV-2, HIV/AIDS, Pregnancy, Maternal Outcomes, Newborn Outcomes, Vertical Transmissions, Breastfeeding, Vaccine, Pandemic, Infectious Disease

## Abstract

**Purpose of Review.:**

Our review aims to compare and contrast Human Immunodeficiency Virus/Acquired Immunodeficiency Syndrome and SARS-CoV-2/COVID-19’s impact on maternal and neonatal outcomes. We have made significant progress in Human Immunodeficiency Virus/Acquired Immunodeficiency Syndrome prevention and treatment over the last few decades. Drawing on empirical evidence with past public health crises can offer valuable insights into dealing with current and future pandemics. Therefore, it is imperative to conduct a comparative analysis of the resemblances and disparities existing between Human Immunodeficiency Virus/Acquired Immunodeficiency Syndrome and SARS-CoV-2/COVID-19.This research endeavor represents a pioneering and all-encompassing examination, aiming to discern and comprehend the parallels and contrasts in the respective impacts of SARS-CoV-2 and Human Immunodeficiency Virus on pregnancy.

**Recent Findings.:**

Based on the current evidence, there is no indication that pregnancy increases women’s susceptibility to acquiring Human Immunodeficiency Virus or SARS-CoV-2. Nevertheless, the state of being pregnant was correlated with the worsening of diseases and their progression. Both Human Immunodeficiency Virus and SARS-CoV-2 pose increased risks of maternal mortality and several obstetric complications, including premature birth and pre-eclampsia. While the vertical transmission of Human Immunodeficiency Virus is well-established, a comprehensive understanding of the vertical transmission of SARS-CoV-2 remains elusive, emphasizing the need for further investigations. Initial data suggest low SARS-CoV-2 vertical transmission rates in the setting of proper preventative interventions and universal screening. A cesarean delivery could reduce the risk of mother-to-child transmission in Human Immunodeficiency Virus-infected women with high viral loads or poor adherence to antiretroviral therapy (ART). However, it did not offer additional protection for Human Immunodeficiency Virus-infected women who adhered to Adherence to Antiretroviral Therapy or those with COVID-19. Human Immunodeficiency Virus and SARS-CoV-2 were linked to neonatal complications such as stillbirth, low birth weight, and neonatal intensive care unit (ICU) admissions. The universal testing of both pregnant patients and neonates is an effective strategy to prevent the spread and complications of both Human Immunodeficiency Virus and SARS-CoV-2. Human Immunodeficiency Virus control largely relies on preventing vertical transmission and medications during pregnancy and postpartum, whereas safety behaviors and vaccines have proven effective in preventing SARS-CoV-2 vertical transmissions.

**Summary.:**

This review aims to compare and contrast the impact of Human Immunodeficiency Virus and SARS-CoV-2 on pregnancy outcomes, vertical transmissions, delivery modalities, neonatal outcomes, and clinical management. SARS-CoV-2 and Human Immunodeficiency Virus were associated with significant obstetric-related complications, making close clinical monitoring and preparation essential. Integration of SARS-CoV-2/COVID-19 management with reproductive health services is crucial to ensuring maternal and neonatal outcomes. Our review is not only the first to establish a groundwork for the current state of knowledge and its clinical implications on this topic, but it also sheds new insights for future research directions.

Comparing Human Immunodeficiency Virus/Acquired Immunodeficiency Syndrome and SARS-CoV-2 in terms of their impact on maternal and neonatal outcomes provides valuable insights despite their differences. Leveraging Human Immunodeficiency Virus/Acquired Immunodeficiency Syndrome research can help understand SARS-CoV-2 effects on pregnancy. Both infections pose risks to pregnant individuals and their fetuses, leading to increased maternal mortality and complications. Identifying common patterns and risk factors can improve clinical management for pregnant individuals with SARS-CoV-2. While a direct observational study for this comparison may not be feasible, comparing with Human Immunodeficiency Virus offers an ethical and practical approach. However, specific studies on SARS-CoV-2 are still necessary to gather detailed data on maternal and fetal outcomes.

## Introduction

Both the human immunodeficiency virus (HIV) and the severe acute respiratory syndrome coronavirus 2 (SARS-CoV-2) infections are affecting millions of people worldwide today.^1^ Since the beginning of the HIV epidemic, 770,000 lives have been lost to HIV/AIDS-related illnesses.^2^ Over the last 40 years, no virus has instilled as much fear as HIV except SARS-CoV-2, a virus that caused COVID-19, which was identified in late 2019. As of May 2021, nearly 170 million people had been infected, and 3.54 million lives had been lost to COVID-19.^3^ We have made significant progress in preventing and managing HIV. By comparing and contrasting two of the deadliest pandemics in history, we can gain valuable insights into managing current and future pandemics.

The first case of HIV infection was reported in 1981, and the first isolation of HIV-1 was achieved in 1983.^4^ Human Immunodeficiency Virus has been identified as the most dangerous among sexually transmitted and blood-borne infections due to its high potential for an outbreak.^5^ The global population of individuals living with HIV exceeds 38 million, with HIV infection during pregnancy emerging as the most common medical complication of pregnancy in some countries.^6^ Mother-to-child transmission (prepartum, intrapartum, or postpartum) via breast milk feeding is a major transmission route for HIV. In contrast, the novel coronavirus disease 2019 (COVID-19) emerged in December 2019 in Wuhan, in the province of Hubei, China. This infectious disease is caused by the severe acute respiratory syndrome coronavirus 2 (SARS-CoV-2) and was officially declared a global pandemic in March 2020.^7^ SARS-CoV-2 is primarily transmitted through infected respiratory fluids, primarily via droplets and aerosols released during coughing, sneezing, talking, singing, or even normal respiration.^8^

Unlike with Human Immunodeficiency Virus, our current knowledge on the impact of COVID-19 on pregnancy and maternal outcomes is largely unelucidated. Pregnant women are especially susceptible to diseases due to physiological changes involving the immune and hematologic systems. During pregnancy, childbirth, and the postpartum periods women undergo dramatic metabolic, hormonal, and physical changes during pregnancy, childbirth, and postpartum periods. Fetuses may be exposed to the viruses during critical periods of fetal development.^9^ Pregnancy is a vulnerable period, and disease exposure can have short- and long-term impacts on the health of mothers and infants. A thorough understanding of the interactions between diseases and the pregnancy process is critical for obstetric management. Through the analysis of the similarities and differences in how HIV/AIDS and SARS-CoV-2/COVID-19 interact with the pregnancy process and outcomes, this review can generate fresh insights into establishing management strategies that are tailored to pregnant patients’ needs.

### Effects of pregnancy on Human Immunodeficiency Virus and SARS-CoV-2 infections

#### Disease Susceptibility during Pregnancy.

Based on the current studies, pregnancy did not alter the risk of contracting Human Immunodeficiency Virus or SARS-CoV-2. Early studies showed that the risk of HIV was not significantly higher among pregnant or postpartum women than nonpregnant females of reproductive age.^10^ However, further investigation showed that the null result was due to behavioral changes during pregnancy. There was a steady increase in the HIV acquisition probability during pregnancy and the postpartum period, suggesting that biological changes during pregnancy and the postpartum period increase HIV susceptibility among women.^11^ Several studies suggested that pregnancy did not increase the susceptibility to SARS-CoV-2 infection.^12–17^ The lessons we learned from HIV suggest that these changes may result from pregnancy-related behavior changes. The biological susceptibility of SARS-CoV-2 among pregnant women requires further investigation. Based on data from universal screening, risk factors for infection among pregnant women include race/ethnicity, insurance status, and geographical location.^18^ While pregnant women do not seem to be at an increased risk for contracting SARS-CoV-2, medical research suggests that universal screenings of pregnant women presenting for check-ups or delivering diseases that may cause obstetric complications may be beneficial.

#### Disease Progression during Pregnancy.

It was shown in a systematic review that pregnant women were more likely than nonpregnant women of reproductive age to be asymptomatic with COVID-19.^13^ Among pregnant women with COVID-19 infections, 95% (95% CI = 45–100%) were asymptomatic, and 59% (95% CI = 49–68%) remained asymptomatic throughout follow-up.^19^ Pregnant women may be overscreened compared to the nonpregnant population. The data highlighted the importance of screening pregnant individuals for COVID-19 to control transmission. For those with symptomatic COVID-19, a report from the Centers for Disease Control and Prevention (CDC), including over 23,000 COVID-19-positive pregnant women and 386,000 women of reproductive age showed that the most common symptoms were coughing (50.3% vs. 51.3%, pregnant vs. nonpregnant, respectively), headache (42.7% vs. 54.9%), myalgia (36.7% vs. 45.2%), fever (36.7% vs. 45.2%), sore throat (28.4% vs. 34.6%), shortness of breath (25.9% vs. 24.8%), and loss of taste or smell (21.5% vs. 24.8%).^12^

Though pregnant women were more likely to be asymptomatic, SARS-CoV-2 could worsen the clinical course of COVID-19 compared with nonpregnant women of the same age. In symptomatic pregnant women, clinical deterioration could occur rapidly. They appeared to have an increased risk of severe disease and death compared to symptomatic nonpregnant women of reproductive age.^12–17^ Reports from the Centers for Disease Control and Prevention showed that COVID-19 pregnant individuals had a higher risk of Intensive Care Unit admission, ventilation, extracorporeal membrane oxygenation (ECMO), and death.^12^ Another prospective study involving 5,183 COVID-19-positive pregnant individuals and 175,905 COVID-19-positive nonpregnant individuals of reproductive age showed similar results: pregnancy increased COVID-19-related risks of Intensive Care Unit admission, pneumonia, and death.^20^ Pregnant women with a prepregnancy underweight condition exhibited a heightened susceptibility to intensive care unit admission (relative risk, 5.53; 95% confidence interval, 2.27–13.44), mechanical ventilation (relative risk, 9.36; 95% confidence interval, 3.87–22.63), and maternal mortality related to pregnancy (relative risk, 14.10; 95% confidence interval, 2.83–70.36).^21^ Other risk factors included older age, obesity, hypertension, diabetes, and other comorbidities.^22,23^ Sadly, non-Hispanic Black individuals accounted for a disproportionate number of deaths.^12^

Similarly for Human Immunodeficiency Virus, according to most studies, pregnancy has been associated with an increased risk of HIV-defining illnesses and HIV-related or all-cause mortality in settings without Antiretroviral Therapy. There was an association between pregnancy and low Cluster Differentiation 4 count (RR = 1.41) in patients who lack Antiretroviral Therapy. Notably, HIV disease progression, AIDS-defining illnesses, and mortality were not accelerated by pregnancy when ART was available. Pregnancy did not adversely affect the natural history of HIV infection in women when ART was available.^24^

Obstetric management and treatments have significantly impacted HIV disease progression during pregnancy and other critical maternal time periods. The results emphasize the importance of early screening and developing treatment strategies tailored to COVID-19/HIV-positive pregnant women to avoid the aforementioned complications. Further research is required to determine whether antiviral medications can treat and safely prevent COVID-19 in pregnant women.

### Effects of Human Immunodeficiency Virus and SARS-CoV-2 infections on pregnancy outcomes

Both Human Immunodeficiency Virus and SARS-CoV-2 were associated with a number of infectious obstetric complications. It is evident from the reviewed literature that pregnant individuals afflicted with SARS-CoV-2 exhibit a notably increased vulnerability to such unfavorable consequences in comparison to their non-infected counterparts.^25^ Women with COVID-19 diagnosis were at a higher risk for severe infections (RR = 3.38; 95% CI = 1.63–7.01).^26^ In the same way, Human Immunodeficiency Virus-positive women had a greater likelihood of urinary tract infections (OR = 3.02; 95% CI = 2.4–3.81), postpartum sepsis (OR = 8.05; 95% CI = 5.44–11.90), and postpartum infection (OR = 3.00; 95% CI = 2.37–3.80).^27^ A meta-analysis showed that Human Immunodeficiency Virus-positive women had a sixfold higher rate of puerperal sepsis (OR = 5.81; 95% CI = 2.42–13.97) and two times the odds of endometritis (OR = 2.51; 95% CI = 1.50–4.21) compared to uninfected women.^28^ Given the high prevalence of infectious complications, particular disinfection protocols should be established to care for these patients.

Most studies have shown an increased preterm birth and emergency delivery associated with severe COVID-19.^9,13,29–34^ Researchers in the Netherlands found that mitigation measures, such as masks, hygiene, and ventilations, against COVID-19, reduced the incidence of preterm and emergency delivery in a recent nationwide quasi-experimental study. With adequate preventative measures, a cohort of 72 neonates born to mothers who tested positive for COVID-19 did not yield any confirmed cases of COVID-19 within 24 hours following birth. Moreover, the investigation revealed that none of the newborns born to COVID-19 positive mothers were identified as COVID-19 positive subsequent to birth.^35^ Limited data have shown that the frequency of miscarriage was not increased with COVID-19.^36–42^

Likewise, there was substantial evidence of an increased risk of preterm delivery of babies born to HIV-positive women.^43^ Additionally, Human Immunodeficiency Virus has been associated with an increased risk of uterine rupture in several studies. According to a study in Thailand, women with HIV had nearly eight times the chance of experiencing prolonged labor than those who are not infected (OR = 7.86; 95% CI = 4.64–13.33).^28^ Human Immunodeficiency Virus-positive mothers also experienced a higher risk of venous thromboembolism (OR = 2.21; 95% CI = 1.46–3.33) and blood transfusions (OR = 3.67; 95% CI = 3.01–4.49).^44^

#### Mortality.

Sadly, Human Immunodeficiency Virus and SARS-CoV-2 were similar in their impacts on maternal mortality. In a multinational cohort study, COVID-19 during pregnancy was associated with significant increases in severe maternal mortality. The risk of maternal mortality was 1.6%, 22 times higher in the group of women with COVID-19 diagnosis (RR = 22.3; 95% CI = 2.88–172).^26^ A recent study in Botswana showed COVID-19 during the time of labor is associated with 3.7% maternal mortality.^45^ According to a conference report, it has been observed that maternal mortality exhibited a pronounced escalation among women who tested positive for COVID, with a nearly 4% maternal mortality rate, representing a substantial 30-fold increase in comparison to pre-pandemic levels.^46^ Similarly, a meta-analysis showed that the risk of pregnancy-related death was eight times higher among HIV-infected women than HIV-negative women (RR = 7.74; 95% CI = 5.37–11.16).^28^ Acquired Immunodeficiency Syndrome has become the leading cause of maternal mortality in some regions since the epidemic started.^47,48^ Among Human Immunodeficiency Virus-infected pregnant and postpartum women, the excess mortality rate was 994 per 100 000. Within regions where Human Immunodeficiency Virus prevalence among pregnant women was 2%, 12% of all maternal deaths were attributable to HIV/AIDS. This prediction rose to 50% in regions with a prevalence of 15%. Human Immunodeficiency Virus was estimated to be responsible for approximately 5% of pregnancy-related deaths worldwide and 25% in sub-Saharan Africa.^49^ Further research is required to develop and assess effective interventions to reduce COVID-19/AIDS-associated maternal mortality.

#### Hemorrhage.

A meta-analysis concluded that Human Immunodeficiency Virus-infected women are twice as likely to suffer an antepartum hemorrhage (OR = 2.06; 95% CI = 0.29–14.92).^28^ Neither SARS-CoV-2 nor HIV was associated with an increased risk of postpartum hemorrhage or retained placenta. The mechanisms behind these associations are unelucidated. Further research on these questions would be a helpful way of developing therapeutics to reduce the risk of hemorrhagic complications. Human Immunodeficiency Virus was not associated with placenta previa or placental abruption.^50^

#### Hypertension.

A meta-analysis of 11 datasets concluded that there was an increased risk of pregnancy-induced hypertension (OR = 1.46; 95% CI = 1.03–2.05) with HIV infection.^28^ However, Human Immunodeficiency Virus infection was not a risk factor for pre-eclampsia or postpartum pregnancy-induced hypertension.^51^ In contrast, compared with no infection, SARS-CoV-2 infection in pregnancy was associated with preeclampsia (OR = 1.33; 95% CI = 1.03–1.73).^9^ In addition, compared with mild COVID-19, severe COVID-19 was strongly associated with preeclampsia. The mechanisms underlying the association between COVID-19 and preeclampsia were unclear, but SARS-CoV-2 might induce renin-angiotensin system dysfunction and vasoconstriction by binding to angiotensin-converting enzyme 2 receptors. Study results found that women with severe COVID-19 who were pregnant developed symptoms similar to preeclampsia and could be identified by biomarkers such as serum-soluble fms-like tyrosine kinase and placental growth factor. The protocol for caring for COVID-19-positive pregnant patients should include frequent blood pressure measurements and adequate control. Infection with SARS-CoV-2 has been shown to generate a proinflammatory state that could lead to preeclampsia and systemic endothelial dysfunction.^52^

Research has shown that pregnancy is a risk factor for severe COVID-19, especially in women with HIV. Close clinical monitoring, emergency preparedness, and clinical precautions are essential for managing these patients. Further research is needed to study the clinical management of obstetric complications in pregnant women with COVID-19.

### Vertical Transmission

An individual could contract Human Immunodeficiency Virus through sexual contact, blood or blood products, donated sperm or organs, or maternal transmission (maternal transmission).^53^ Over 70% of infections were caused by heterosexual transmission, and approximately 90% of cases in children were caused by mother-to-child transmission.^54^ Annually, over 600,000 children were infected through vertical transmission. In the absence of treatment, the risk of mother-to-child transmission of the human immunodeficiency virus (HIV) was between 25–30% during pregnancy, delivery, and breastfeeding.^55^ Vertical transmission in the United States has become less than 2% since Human Immunodeficiency Virus testing, counseling, antiretroviral medications, and deliveries before the onset of labor have been implemented.^2^

Mother-to-child transmission could occur via breast milk feeding during prepartum, intrapartum, or postpartum. Based on fetal tissue studies, it was concluded that most of the utero vertical transmissions occurred during the third trimester.^56^ HIV broke down the integrity of the placenta, leading to micro-transfusion of the viremic maternal blood that crossed the placenta to the fetus.^57^ Human Immunodeficiency Virus in the form of Ribonucleic Acid (RNA) has also been identified in breast milk, indicating the potential involvement of the intestines or tonsils as portals of entry for HIV in breastfed infants. It is postulated that viral infections could infiltrate the infant’s body through compromised intestinal epithelial integrity or weakened cellular tight junctions.^58–61^ Noteworthy findings reveal that infants who acquire Human Immunodeficiency Virus through breastfeeding exhibit elevated levels of lipopolysaccharide, implying a possible facilitative role of compromised intestinal integrity in the transmission process.^62^

In contrast to the established evidence of Human Immunodeficiency Virus vertical transmission, a clear understanding of vertical transmission of SARS-CoV-2 (in utero, intrapartum, early postnatal period) is still lacking. It is observed that the incidence of vertical transmission of SARS-CoV-2 appears to be infrequent according to existing reports. However, it is crucial to acknowledge that despite this rarity, potential hazards to fetal wellbeing persist, warranting further attention and investigation.^63^ Only a few cases of congenital infection have been reported in the setting of third-trimester maternal infection.^64^ According to a systematic review of infants born to 936 COVID-19-infected mothers, 2.9% tested positive for COVID-19 shortly after birth. Furthermore, 3/82 neonatal serologies were positive for SARS-CoV-2 immunoglobulin M (IgM).^65^ The research findings suggest that there is a notable correlation between maternal SARS-CoV-2 infection and a twofold rise in the likelihood of infant infection (Hazard Ratio [HR] = 2.31, 95% Confidence Interval [CI]: 1.08–4.94). It is worth noting that a relatively small proportion of the participants, specifically 13% of the mothers and 33% of the infants, exhibited symptoms related to COVID-19. Nevertheless, it is noteworthy that none of the participants experienced severe manifestations of the disease or succumbed to it.^66^ SARS-CoV-2 had rarely been detected in vaginal secretions or amniotic fluid.^67^ However, the feces excretion associated with labor pushing could contain the virus.^68,69^ In terms of vertical transmission of SARS-CoV-2 during breastfeeding, a New York City study of 82 infants of 116 mothers who had COVID-19 reported no postnatal positive SARS-CoV-2 infection after breastfeeding.^37,70^ It appears that newborns receive passive SARS-CoV-2 immunity through antibody transfer via the placenta and from the breast milk following natural infection.^71^ This encouraging result suggests an efficient transplacental transfer of maternal antibodies. Further studies regarding the impact of these antibodies on infant immunity would be worthwhile.

As a result of universal screening and precautions at birth, in addition to early initiation of Antiretroviral Therapy, Human Immunodeficiency Virus vertical transmission has been successfully reduced. In the context of the French Perinatal Cohort comprising 14,630 women who were HIV-positive and underwent deliveries between 2000 and 2017, it was observed that both theHuman Immunodeficiency Virus in the form of Ribonucleic Acid level at the time of delivery and the commencement period of Antiretroviral Therapy (ART) initiation exhibited separate and significant associations with the likelihood of perinatal transmission of HIV. This finding suggests that both factors independently play a role in influencing the risk of transmitting the virus from mother to child during the perinatal period.^72^ Consequently, screening and appropriate precautions should be used to prevent vertical transmission of SARS-CoV-2. The investigation will encompass an analysis of the clinical manifestations of COVID-19 in pregnant individuals. Moreover, an evaluation will be conducted to determine the prevalence of infection during pregnancy, alongside an exploration of the risk factors contributing to maternal and neonatal morbidity and mortality associated with SARS-CoV-2 infection. Additionally, the potential for motherto-child transmission of SARS-CoV-2 will be scrutinized. The diagnostic procedure for SARS-CoV-2 infection screening will involve the utilization of Polymerase Chain Reaction diagnosis.^73^ Further research is required to determine the vertical transmission of SARS-CoV-2.

### Effects of Human Immunodeficiency Virus and SARS-CoV-2 infections on the route of delivery and vice versa

Significant variations exist in the recommended delivery approaches for mothers infected with SARS-CoV-2 and those with Human Immunodeficiency Virus. In the case of HIV-infected women with high viral loads or poor adherence to Antiretroviral Therapy, the possibility arises that a cesarean delivery performed prior to labor initiation and before the rupture of membranes could potentially mitigate the risk of mother-to-child transmission. During the delivery process, vertical transmission of HIV may transpire if maternal blood and secretions come into contact with the infant’s mucosal membranes. Notably, in the absence of antiretroviral treatment, prolonged membrane ruptures exceeding four hours have been linked to an escalated likelihood of transmission.^74^ However, it should be noted that within the context of Antiretroviral Therapy adherence, the application of a cesarean section offers limited additional benefits in preventing mother-to-infant HIV transmission.

Neonatal COVID-19 infection risks were not reduced by cesarean delivery.^75,76^ Under the current guidelines, an asymptomatic or mild COVID-19 status did not indicate that these patients needed to change the delivery route. Based on a small study of 37 cesareans and 41 vaginal deliveries, cesarean delivery was associated with an increased risk for clinical deterioration (aOR = 13; 95% CI = 1.5–121.9; p = 0.02) in COVID-19 patients.^77^ Cesarean delivery was performed for patients with severe or critical COVID-19. Even if labor induction is safe among intubated patients, it could be impractical due to the lack of specialized equipment and personnel in the ICU.^78,79^

Based on evidence, managing Human Immunodeficiency Virus among pregnant women has shown that vaginal birth is preferred when diseases are appropriately managed and appropriate precautions are taken. Even if future studies confirm the possibility of vertical transmission during delivery, it will not justify cesarean delivery because of the increased risk to the mother and the lack of beneficial effects to the newborn since reports of COVID-19 infection in newborns have been generally mild.

### Effects of Human Immunodeficiency Virus and SARS-CoV-2 infections on neonatal outcome

Compared with no infection, SARS-CoV-2 infection in pregnancy was associated with stillborn (OR = 2.11; 95% CI = 1.14–3.90).^9^ SARS-CoV-2 infection for women during the time of delivery is associated with 5.6% of stillbirth.^45^ Research on the causes of stillbirth was limited. According to a conference report, it has been observed that infants born to women who tested positive for COVID-19 exhibited a higher incidence of adverse birth outcomes, with a notable 5.5% risk for stillbirth, representing a 2-fold increase compared to the general population. Furthermore, among COVID-positive women with HIV, the risk for experiencing the most adverse birth outcomes was found to be the highest.^46^ Lupus anticoagulant was monitored as part of stillbirth evaluation, and it has been seen transiently in COVID-19 patients.^30,80–82^ Similarly, the rate of stillbirth among HIV-positive pregnant women is 1.67 times higher than HIV-negative pregnant females.^83^

Based on preliminary examination, it was observed that maternal and neonatal mortality rates surged in the three provinces of South Africa amidst the COVID-19 pandemic, while stillbirth rates showed no significant change.^84^ More than 95 percent of newborns born to SARS-CoV-2-positive mothers were in good health when they were born. Most infants diagnosed with SARS-CoV-2 were either asymptomatic or had a mild presentation. Postnatal transmissions were believed to be responsible for most cases.^13,34,75,85–87^ To date, COVID-19-related congenital anomalies have not been reported.^88^ Neonatal mortality and the length of neonatal hospital stay were not altered by maternal COVID-19 status. A systematic review found that the neonatal death rate was similar among positive and negative maternal SARS-CoV-2 groups.^75^ Neonatal outcomes are the worst when they are exposed to both SARS-CoV-2 and Human Immunodeficiency Virus.^45^

Compared with mild COVID-19, severe maternal COVID-19 was strongly associated with low birth weight and Neonatal Intensive Care Unit admission.^9^ According to a Swedish study, infants born to SARS-CoV-2-positive mothers had a slight increase in any respiratory disorder (OR = 1.42; 95% CI = 1.07–1.90) and hospital admissions (OR = 1.47; 95% CI = 1.26–1.70), compared to those born to uninfected mothers.^32^ In a study, in neonates born to women with SARS-CoV-2 infection, there was an elevated likelihood of admission to a neonatal care unit following birth (seven studies; n=7637; relative risk [RR] 1.86, 95% confidence interval [CI] 1.12 to 3.08). Additionally, these infants exhibited a higher probability of being born preterm (seven studies; n=6233; RR 1.71, 95% CI 1.28 to 2.29) or moderately preterm (seven studies; n=6071; RR 2.92, 95% CI 1.88 to 4.54), as well as being born with low birth weight (12 studies; n=11,930; RR 1.19, 95% CI 1.02 to 1.40).^89^ SARS-CoV-2 infection might also cause exaggerated systemic inflammatory responses involved in the pathogenesis of preterm birth, or a suboptimal fetal growth and development environment.^90^ Placental fetal vascular mal-perfusion has been found in placental histopathologic findings in patients with COVID-19 at delivery, which might contribute to fetal growth, stillbirth, and preterm birth.^91^ The confluence of clinical observations, placental examination, and immunohistochemical alterations convincingly indicates that maternal SARS-CoV-2 infection during the second trimester, accompanied by placentitis, elicited a pronounced inflammatory reaction and oxidative stress damage to the fetoplacental complex, which consequently impacted the fetal brain. Furthermore, the identification of SARS-CoV-2 within the deceased neonate’s brain raises the potentiality that direct infection of the fetal brain by SARS-CoV-2 may have substantially contributed to the perpetuation of cerebral impairment.^92^ Three cohort studies of pregnant COVID-19 patients have shown that the risk of preterm birth was increased among patients with severe COVID-19.^20^ The impact of maternal COVID-19 infection on the fetal immune and nervous systems has been observed. While the SARS-CoV-2 virus itself does not directly invade the fetus, the exposure to an inflammatory milieu during maternal infection can incite activation of the fetal immune system. This interaction between maternal and placental/fetal immune activation is believed to exert an influence on the developmental processes of the fetal nervous system.^93^ Further research should be conducted exploring the physiological mechanisms and effective clinical interventions.

Neonates born to these Human Immunodeficiency Virus-positive mothers were at increased risk of prematurity and intrauterine growth restriction. A meta-analysis of prospective cohort studies showed that maternal HIV infection is associated with an increased risk of preterm birth (RR = 1.50), low birthweight (RR = 1.62), and small for gestational age (RR = 1.31).^28^ Retrospective cohort studies also suggested an increased risk of term low birthweight (RR = 2.62; 95% CI = 1.15–5.93) and preterm low birthweight (3.25; 95% CI = 2.12–4.99).^28,55^ No association was identified between maternal HIV infection and preterm birth, very low for gestational age, birthweight, miscarriage, or neonatal death, although few data were available for these outcomes. Consequently, further research is required to determine these HIV-related neonatal outcomes comprehensively.

### Management Approaches of Pregnant Women with Human Immunodeficiency Virus and SARS-CoV-2

#### Human Immunodeficiency Virus/COVID Management during Pregnancy-- Testing and Medication

In developed countries, the low vertical transmission rate of HIV can be attributed mainly to HIV testing and counseling, antiretroviral medications, deliveries before the onset of labor, and the disallowance of breastfeeding.^2^ Universal Human Immunodeficiency Virus screening on pregnant patients is implemented in many countries. In pregnant women, the HIV viral load and CD4+ T cell count are closely monitored through blood tests. Antiretroviral Therapy is indicated for all patients with HIV. If the Antiretroviral Therapy regimen can adequately control the patient’s viral load during pregnancy, the patient may continue with the same treatment. The early initiation of antiretroviral therapy during pregnancy is associated with a reduced viral load at the time of delivery. To prevent the rest of Human Immunodeficiency Virus vertical transmission during delivery, zidovudine should be administered before delivery. Zidovudine is administered to infants born to HIV-positive mothers during the first four to six weeks to prevent vertical HIV transmission.

Given the success of Antiretroviral Therapy in reducing vertical transmission of Human Immunodeficiency Virus, further research should be conducted on the use of antiviral medications to prevent and treat COVID-19-positive pregnant women. Pregnant women who are infected with COVID-19 present unique challenges in terms of management and treatment. However, promising preliminary findings suggest that sotrovimab may be an effective and well-tolerated treatment option for pregnant COVID-19 patients.^94^ In this study, a cohort of 35 pregnant respondents was investigated to evaluate the utilization of nirmatrelvir-ritonavir during pregnancy. The results revealed that 51.4% of the participants were prescribed this medication, and 34.3% actually took it as per the prescription. Among those who ingested nirmatrelvir-ritonavir, a noteworthy 91.7% reported experiencing dysgeusia, while 50.0% exhibited rebound symptoms (comprising 50.0% positive test result and 33.3% symptom recurrence).^95^

#### Human Immunodeficiency Virus and COVID-19 Management -- Neonatal Testing

Polymerase Chain Reaction tests for Human Immunodeficiency Virus are recommended to determine the HIV status of infants born to HIV-positive mothers. As Human Immunodeficiency Virus antibodies are transmitted from the mother to the child, HIV antibody tests are not accurate in determining the HIV status of an infant. Similarly, infants born to mothers with suspected or confirmed COVID-19 should be tested for SARS-CoV-2 RNA using reverse transcription-polymerase chain reaction (RT-PCR).

#### COVID-19 Management during Pregnancy -- Testings and Behavioral Precautions

In the wake of the Human Immunodeficiency Virus and other infectious disease prevention lessons, many hospitals now routinely screen prospective mothers presenting for childbirth for SARS-CoV-2. Data regarding mother-to-infant transmission in the postnatal period have been reassuring when appropriate precautions are taken. Mothers who wear surgical masks and follow proper hand hygiene guidelines can safely perform skin-to-skin care and breastfeed. In a study of 116 SARS-CoV-2–positive mothers who breastfed their 120 newborns, all newborns tested negative for SARS-CoV-2 and were asymptomatic. In this study, the infants roomed with their mothers in a closed isolette, and mothers used a surgical mask and careful hand and breast hygiene before breastfeeding and other interactions with the infant.^70^

#### COVID-19 Management during Pregnancy-- Vaccines

Despite the higher risk of COVID-19 complications, pregnant individuals were not allowed to participate in the clinical trials of the COVID-19 vaccines, as is often the case with new medications and vaccines. Pregnant women can receive nearly all vaccines if their benefits outweigh their risks, except for live-attenuated vaccines, which are contraindicated due to the theoretical risks of the virus crossing the placenta and infecting the fetus. A small number of prospective cohort studies and data obtained from pregnant women who were vaccinated indicate that there are no harmful effects.^96^ An analysis of a retrospective cohort study revealed that maternal infection by SARS-CoV-2 was reduced by BNT162b2 mRNA vaccination during pregnancy compared with not vaccinating (adjusted hazard ratio 0.22; 95% CI = 0.11–0.43).^44^ The presence of protective antibodies has been documented in cord blood 15 days following maternal mRNA vaccination.^97^ Vaccine-elicited antibodies have been detected in the cord blood and breast milk. Pregnant and nonpregnant vaccinated women developed cross-reactive immune responses against the B.1.1.7 and B.1.351 SARS-CoV-2 variants of concern.^98^ The results demonstrate neutralizing antibodies in both cord blood and breast milk, suggesting the possibility that newborns may be protected by maternal vaccination. In light of the benefits of breastfeeding and the safety data of vaccines, the Centers for Disease Control and Prevention (CDC), the American College of Obstetricians and Gynecologists (ACOG), and the Society for Maternal-Fetal Medicine (SMFM) all provide reassurance regarding initiating or continuing breastfeeding in a newly vaccinated individual. The next step should determine the optimal vaccination schedule to deliver breast milk antibodies to neonates.

According to the scholarly publication titled “Effects of COVID-19 vaccination during pregnancy on SARS-CoV-2 infection and maternal and neonatal outcomes: A systematic review and meta-analysis,” there was no discernible evidence to suggest an elevated risk of adverse outcomes, such as miscarriage, gestational diabetes, gestational hypertension, cardiac complications, oligohydramnios, polyhydramnios, unassisted vaginal delivery, cesarean delivery, postpartum hemorrhage, gestational age at delivery, placental abruption, Apgar score at 5 minutes below 7, low birthweight (<2500 g), very low birthweight (<1500 g), small for gestational age, and neonatal fetal abnormalities, among pregnant individuals who received COVID-19 vaccination.^99^

In accordance with the scholarly discourse presented in the research article titled “COVID-19 and Pregnancy: Clinical Outcomes, Mechanisms, and Vaccine Efficacy,” the United States Food and Drug Administration (FDA) advocates the administration of existing COVID-19 vaccines to pregnant individuals as a viable and secure measure. The purpose behind this recommendation is to mitigate the incidence of severe pregnancy-related complications arising from SARS-CoV-2 infection.^63^

There is still a great deal of vaccine hesitancy amongst pregnant individuals. Merely 55% of the total participants (12 out of 22 participants) underwent the full regimen of primary vaccination, with distribution among vaccine types as follows: 46% received mRNA-1273, 46% received BNT162b2, and merely 8% received JNJ-78,436,735. No individuals within the cohort received a booster dose during the study period.^94^ The results of a small cohort study that included 30 pregnant and 16 lactating women demonstrated that all participants were immunogenic after receiving COVID-19 messenger RNA.^100^

## Conclusion

In this review, we have analyzed the similarities and differences between Human Immunodeficiency Virus/Acquired Immunodeficiency Syndrome and SARS-CoV-2/COVID-19’s impacts on maternal and neonatal outcomes. Our manuscript is the first comprehensive analysis of the impact of HIV vs. SARS-CoV-2 on pregnancy. Due to the novelty of the SARS-CoV-2 virus, the mechanistic relationship behind the contraction of the virus and most pregnancy complications remains largely unelucidated. Understanding the association and mechanistic causes is crucial to establishing management strategies to prevent viral transmissions and minimize complications. Pregnancy is one of the most vulnerable and critical periods. Through the examination of the parallels and differences between HIV and SARS-CoV-2, the review sheds light on the urgent need to examine the association of viral infection and pregnancy complications, the impact of co-infection of HIV and SARS-CoV-2 during pregnancy, the effectiveness of prenatal and postnatal management to minimize vertical transmissions and obstetric complications. Given the substantial excess of pregnancy-related complications associated with SARS-CoV-2 and HIV, the review highlights the importance of universal screening, close clinical follow-up, and adequate precautions. Integrating SARS-CoV-2/HIV treatment with clinical reproductive management is crucial to ensure the safety of mothers and newborns.
